# Behavior of Internal Customer in Family Business: Strategies and Actions for Improving Their Satisfaction

**DOI:** 10.3389/fpsyg.2017.01266

**Published:** 2017-07-25

**Authors:** Santiago Gutiérrez-Broncano, Pedro Jiménez-Estévez, María del Carmen Zabala-Baños

**Affiliations:** Business Administration, University of Castilla-La Mancha Talavera de la Reina, Spain

**Keywords:** family business, internal customer, socio-emotional wealth, human resource management, human organizations

## Abstract

Determining the relevant aspects of family businesses (FBs) that make them increasingly competitive is the main objective of researchers in this field. Despite this, there is little empirical literature on the behavior of the internal customer (IC) in FBs or how businesses increase their satisfaction. Basing our work on psychological theories and with both quantitative and qualitative information from 31 semi-structured interviews, this work establishes certain characteristics of the ICs of the FB and proposes a series of guidelines for increasing their satisfaction, thereby facilitating the continuity of this type of business. FBs that are able to understand that the motivation of their ICs is more important than other qualities, and that this requires a more comprehensive management will be able to get sustainable competitive advantages in the future.

## Introduction

Research in the area of family businesses (FBs) has grown exponentially in recent years. This is partly due to recognition of their economic impact, generation of employment, and contribution to economic development (Colli, 2003; Zahra, 2005; Zahra et al., 2008; Dawson, 2012), but also due to increased interest of researchers in this area, mainly due to the special characteristics of this type of business and also the results that they achieve (Sharma et al., 2007; Stewart and Miner, 2011). Recent evidence suggests that FBs significantly outperform non-FB (NFBs) in sales and assets (McConaughy et al., 2001; Anderson and Reeb, 2003; Anderson et al., 2003; Weber et al., 2003), maintaining a higher revenue for the first generation (Weber et al., 2003) and possibly firmer longevity (De Geus, 1997; Mackie, 2001).

Scholars have tried to explain this advantage with several reasons that Dawson (2012) groups in three levels: group level (social relations), firm level (familiness), and individual level; but no conclusive result has been obtained. This study is focused on the individual level, as it is where we find the least literature, more specifically viewing the members of FBs from a marketing perspective, as internal customers (ICs).

Today, businesses must get their competitive advantage through the effective use of all resources, especially those that are more difficult to imitate, to achieve more sustainable distinguishing advantages. Although the literature has paid close attention to external customers, it has not done the same toward ICs (Stanley and Wisner, 1998). Many writers place both types of customer on the same level (i.e., Stanley and Wisner, 1998; Chang and Huang, 2010; Jun and Cai, 2010) due to the degree of difficulty involved in both their ability to imitate and their effective management.

Internal marketing (IM) seeks to attract, develop, demonstrate and retain qualified employees (Berry and Parasuraman, 1991) and these employees are seen as ICs (Foreman and Money, 1995). It has traditionally been understood as a management philosophy focused on effective internal relationships between individuals at all levels of the organization (Keleman and Papasolomou-Doukakis, 2004). However, today the theory of IM shows us how to make employees more satisfied, motivated and prepared to act better toward external customers (Joseph, 1996). To do so, the company has different tools that it must promote, such as effective internal communication, necessary training and development, encouragement of work teams, development of a well-defined organizational structure, and recognition and empowerment of employees (Bennett and Barkensjo, 2005). Among the literature on Strategic Human Resource Management, we also find empirical evidence of the impact that management oriented toward IC satisfaction has on the creation of competitive advantage and improved performance of the organization (Ullah and Yasmin, 2013). Little is known with regard to the process through which this value is created (Wright et al., 2003) and knowledge of the way in which FB achieves this objective is practically non-existent (Reid et al., 2002).

Traditionally, the owners of FBs are often criticized for employing less-skilled members of the family to carry out high responsibility functions (Chrisman et al., 2004), but what is true is that the IC has characteristics that make them especially unique, belonging to two systems at the same time: that of the business and that of the family. This leads to behavior which in certain cases favors the achievement of advantages for the company, but in others may be harmful (Nicholson, 2005b). Analyzing what is the most effective behavior of the IC in FBs and determining what set of strategies and actions carried out within businesses which help to increase the level of IC satisfaction is the main objective of this research work. There is empirical evidence of how the satisfaction and commitment of employees serves to increase effort and show more energy and enthusiasm from employees at work, thus improving performance and customer service (Alfes et al., 2013; Menguc et al., 2013; Karapete and Demir, 2014; Paek et al., 2015). Achieving IC satisfaction, we increase both motivation and business performance.

This orientation toward IM includes three essential aspects (Ruizalba et al., 2015): collecting information, internal communication, and responding to detected needs. Following these steps, this work begins firstly with the compilation of information on the characteristics of the IC in the FB, allowing their behavior to be known and better predicted, and secondly analyzing the main strategies that FBs carry out both for conveying and for implementing different actions that will help to achieve greater satisfaction of ICs. Finally, research findings, discussion, academic implications, business practices, limitations, and future research lines are also presented.

## Literature Review

### Importance of Internal Customer in Family Business

To be able to understand the importance of the IC within the FB we must firstly know what has been traditionally understood by FB. Scholars have understood FB as a combination of two systems: family and business, and the overlap between them (Habbershon et al., 2003). The definition has always been related with regular interaction between members of the family and the control of ownership, and this generates unique characteristics, which may in some cases generate competitive advantages, or conversely cause risks and vulnerabilities to the business and family (Nicholson, 2005a).

The definition of FB has focused on some combination of the four components of the family’s involvement in the business, which are ownership, governance, management and transgenerational succession (Chua et al., 1999). If we search for a theoretical definition, we can understand FB as the family’s influence over the strategic management of a firm (Davis and Tagiuri, 1989), or as the intention of the family to retain control (Litz, 1995), or potentially as the unique, inseparable, synergetic resources, and capabilities arising from family involvement and interactions (Habbershon et al., 2003).

The family’s involvement favors the notion that the FB often possesses unique characteristics and sources of competitive advantage compared to the non-FB. Among its sources, Zahra et al. (2008) include reduced agency costs through owner control, longer investment time horizons, increased commitment to intergenerational wealth, stronger investment time horizons, lowered transaction costs due to a higher level of trust, and a less formal or more flexible organizational structure (Geeraerts, 1984). All of these resources and capabilities related with family involvement and interactions are referred to as “familiness.” Familiness is defined as the idiosyncratic set of resources and capabilities resulting from the interaction between two systems: family and business (Habbershon et al., 2003; Zahra, 2005).

Familiness is believed to be of such importance due to it reflecting the positive aspects of overlapping subsystems of family and business (Habbershon et al., 2003), and it is often used as a unique element that can differentiate family and NFBs (Pearson et al., 2008). Nevertheless, these interactions between family and business do not always result in a positive contribution to the FB. Recent research has shown that family influences may have negative effects on the FB as well (i.e., Kellermanns et al., 2012).

When we focus on analyzing the characteristics of the IC, we realize that this refers to the human capital of the FB and find that members of the family have a greater degree of commitment and cooperation compared to non-family employees (Barnett and Kellermanns, 2006). This human capital is a vital resource for the strategy of organizations (Colbert, 2004), but we cannot forget that the knowledge, skills and abilities of individuals are not enough if we want to create value for an organization (Wright et al., 1994; Colvin and Boswell, 2007).

Following resource based-view (Barney, 1991), human capital is the most valuable and difficult to imitate resource, as it is the result of a complex social structure established over time, essentially in FBs. Human capital has included knowledge, skills and abilities, but recently, Hoy and Sharma (2009) have included an intellectual and psychological dimension. In the context of FBs, human capital includes factors such as commitment and emotions as well as integrity, compassion and forgiveness of family members (Puhakka, 2002; Dawson, 2012), with all these factors being referred to as “family human capital.”

For this reason, Kidwell et al. (2012) consider increased attention to employees or ICs in FBs to be necessary. They are important not only for the fast growth of FB but because FBs have two additional characteristics, which affect how they manage their ICs, the presence of family and non-family members, as well as the family influence (Botero and Litchfield, 2013).

To better understand the characteristics of the IC in the FB, Pieper (2010) proposes making greater use of psychological theories, which will improve understanding of the subject. Concepts such as motivation, power and authority, obedience, groupthink, group cohesion, leadership and commitment must be taken into account upon implementing a strategy adapted to FB to improve IC satisfaction.

### Characteristics and Behavior of Internal Customer in Family Business

Traditionally, research carried out on FB has been undertaken with a smaller theoretical basis, mainly focused on agency theory and resource-based view (Chrisman et al., 2010). In whole, agency theory has been somewhat criticized, as it does not exhaustively consider cooperative behavior between the members of the family (Eddleston and Kellermanns, 2007). However, as we previously stated, in recent years, authors have appeared who defend the need to apply psychological concepts to understand certain essential aspects in FBs (i.e., Björnberg and Nicholson, 2007; Pieper, 2010). These theories incorporate aspects such as motivations, emotions, knowledge, personal bonds, expectations and family relationships, which are priorities in the FB (Schulze et al., 2001) and which have not been deeply analyzed. Individual psychology focuses its interest on perceptions, emotions and motivations, while social psychology helps to understand how all of these variables are influenced by the presence of other people (Pieper, 2010).

When we focus on IC of FB, we must be aware that they have very specific characteristics, essentially being members of the same family. These individuals are interested not only in aspects related with their obligations and duties to the company and derived from their job, but also show interest in aspects such as, for example, loyalty, reputation, security, love and affection, cooperation, etc. (Rothausen, 1999). Recently, the theory of socioemotional wealth (SEW) has moved in this direction. It is established that FBs have a preference for objectives which are not exclusively economic, but focused on the maintenance of family influence and control, the identification of family members with the company, the strengthening of social bonds, and even the tendency to increase the emotional attachment and affection of the family toward the business through dynastic succession (Berrone et al., 2012), or even family harmony (Zellweger and Astrachan, 2008).

A study carried out by Björnberg and Nicholson (2007) shows us the existence of both positive and negative emotions between members of the FB (Schulze et al., 2001) and expands its study to analyze the different motivations existing in them.

This leads us to suggest that FB is in many ways more consistent with human design than other NFBs. Nicholson (2008, p. 106) even goes so far as to define it as “the primary economic unit in the history of our species.” The extreme overlap of family and business allows the IC’s need for dependence and acquisition of status in the same measure, mainly due to the possibility of intergenerational transfer, the maximum responsibility generated with the economic unit of the family and the commitment of all members, whether family members or not. This has a direct effect on the IC, as they feel free to express their emotions, trust in their lasting bonds, their personal and organizational objectives being closely aligned with ownership, thus achieving greater satisfaction.

Nicholson (2008) made one of the main studies of the few existing on the characteristics of the members in FB. Which based on evolutionary psychology establishes different characteristics, which in certain cases differs from existing literature on FBs.

There are various types of motivation, which the IC generally has. For example, those who are intrinsically motivated mainly seek their own interests, enjoyment or satisfaction, while those who are extrinsically motivated do so in pursuit of a certain goal, which is usually economic (Abelson et al., 2004). This extrinsic motivation may generate cooperative behaviors, but in a short time period. However, in FB there is a strong element of altruism (Karra et al., 2006). This altruistic behavior is not only between members of FB, but also toward the exterior of the business, affecting the communication, participation and between satisfaction of IC (Van den Berghe and Carchon, 2003). Aspects such as owning their own business and the possibility of transferring it to the next generation, feeling proud of their surname and working as a family have been found to be reasons as or more important than financial reward (Astrachan and Jaskiewicz, 2008).

Furthermore, due to the interaction between the two systems, the members of FBs develop a tacit knowledge and greater social intelligence (Sirmon and Hitt, 2003). This stock of social capital includes personal contact in the business, networks, the ability to remember faces, develop empathy, and analyze the main motivations of others (Lee et al., 2003). All of this enables them to perceive betrayal or the breaking of agreements more reliably, and even detect opportunistic behavior (free rider).

Compared with what is traditionally thought of teams work, in FBs there is a greater diversity based on this affirmation of what Nicholson (2008) refers to as the “genetic lottery,” which provides each member of the family with unique attributes and different potential (Ilies et al., 2006). This makes teams in FBs more heterogeneous than the majority of teams formed in NFBs, which use standardized criteria when selecting members.

With regard to management style, Nicholson (2008) differentiates between leadership gets by inheritance compared with leadership by selection. Their behavior differs, inheritance leaders know and accept their limitations better, leading them the propose new forms of leadership (i.e., co-leadership) and to see themselves as servants of the business and responsible for the family legacy, and serving to attract the loyalty of others. Meanwhile, selected leaders perceive greater capability than the rest of their colleagues.

The existence of blood relationships between the different members of the FB has been criticized by the literature due to the risk of falling into nepotism, this being understood as the tendency to favor members of the family over non-members (Kruger, 2003; Neyer and Lang, 2003). However, this characteristic may also be seen to generate advantages when cooperating and generating confidence between the different members of the FB (Arregle et al., 2007). This situation favors the creation of large clans that share common interests and creates an ideal environment for generating unity, collaboration and cooperation between its members.

Conflicts have also been seen by literature on FB as a great threat (Zellweger and Astrachan, 2008). However, we must differentiate between types of conflict. For example, certain levels of conflict (slight or moderate) in tasks or processes incentivise more innovative behavior and improve results (Pieper, 2010). Conversely, in FB, the conflicts of greater risk are those, which are interpersonal and inter-group, generated for different reasons, which lead to inefficient work (Egea Romero, 2014). There are different types of interpersonal conflicts in FBs: between parents and children, between siblings, between parents, and between cousins. The conflict between parents and children arises because while one group protects its genetic investment, the other pursues its own autonomy. When there is conflict between siblings it is due to them competing for what they consider limited resources, whether economic or emotional (Hertwig et al., 2002). However, the conflict that is the greatest danger in the FB is between parents, due to its danger of divorce, and because in the majority of cases it puts the survival of the business in serious danger (Davis and Harveston, 2001). Despite all these potential conflicts, when they are overcome, it leads to faster decision-making, more effective communication and greater loyalty and commitment of members (Tagiuri and Davis, 1996).

Finally, with regard to more informal and more irrational behavior that characterizes FBs, this is a more natural human behavior. Aguado (2005) describes emotion as being older than cognition, and the organism trusting in it more than in rationality. Emotions represent our first contact with the reality that we perceive, determining the direction of our responses, and it is later that we give a cognitive explanation to these emotional sensations. Therefore, the human mind does not operate like a computer, and is instead imprecise, illogical, selective and intuitive. It is organizational rationality that attempts to correct is through systems and routines. This is the main reason why we find structures that are more organic in FBs, for integrating both systems in a more natural way (Denison et al., 2004). Harris and Reid (2008) found that FBs use four times less work commissions and formal meetings compared with indirect communication with their employees than non-FBs.

### Strategies and Actions for the Improvement of Internal Customer Satisfaction

Research directly related with strategies and actions for the improvement of IC satisfaction is scarce and fragmented. Furthermore, the lacking empirical evidence is inconclusive and even has contradictory results. For this reason, and based on literature on strategic human resource management and on SEW theory, we establish a series of actions of FB that may improve the satisfaction of the IC.

In the previous section, it has been demonstrated how IC in FB has different characteristics and behaviors from other types of customers. When business shows an interest in the satisfaction of the IC, actions must be established that considers these characteristics, as it is a member of the business that also belongs to the family, and this make the process more complex, requiring it to be dealt with in a comprehensive and holistic way.

Strategic management of human resources establishes the main objective of attracting, developing and retaining the best human capital for organizations. This is achieved through practices such as recruitment, selection, training and development, performance evaluation, remuneration system, participation and communication (Wright et al., 2003; Shih et al., 2010). This set of practices seeks performance through the participation and satisfaction of the IC (Guthrie et al., 2002), and has been referred to in several ways: high performance work system (Huselid, 1995), high involvement work system (Edwards and Wright, 2001) and high commitment work system (Arthur, 1992). Its main characteristic is that, among other objectives, greater satisfaction and commitment is obtained among ICs (Ostroff and Bowen, 2000).

Firstly, the strategies and actions for attracting and selecting employees has sought alignment between the employee and the job to fill (Gomez-Mejia et al., 2012). However, in the FB it seems more logical, given that it has a double objective (economic and emotional), to think that it seeks a greater alignment with the organization, rewarding the coinciding of values of the individual and the organization. In this way, the IC will find greater satisfaction not only in what they do (task), but in how they do it (process) and with who they do it (team). It is necessary to introduce tools that make the IC enjoy their work and feel emotionally attached to it Paek et al. (2015).

In the same way, actions focused on training are oriented to the employee learning specific skills and improving their performance. While development intends for the employee to acquire a large number of personal skills and competencies, which in the majority of cases are acquired through different experiences that they receive from different jobs through which they pass (Gomez-Mejia et al., 2012). FBs consider that IC satisfaction is greater when the employee achieves their own professional development, and less so due to alignment with the job (Cruz et al., 2011). Following the model of job characteristics developed by Oldman and Hackman (2010), which is widely accepted in the field of organizational psychology, it is established that skill variety, task identity, task significance, autonomy and feedback are the essential factors for achieving greater IC satisfaction, better performance, greater commitment to the FB, and thus obtaining what is referred to as “psychological ownership” (Oldman and Hackman, 2010).

Additionally, the evaluation of performance and the remuneration system are very useful actions for aligning the objectives of each individual with the objectives of the organization (Subramony, 2009). However, FB does not specify as many measures for achieving this alignment, as both executives, employees, and all members of the business in general seek the best interests of shareholders, who in the majority of cases are themselves or a close relative. The payment of bonuses in NFBs is up to 10% greater than in FBs (Anderson and Reeb, 2003). In this way, it is logical to think that FBs do not also require an evaluation and remuneration system as sophisticated as NFBs to achieve the same result (Cruz et al., 2011).

Finally, all organizations specify an effective flow of communication, as this is crucial for the proper operation of any kind of business (Riggio and Lee, 2007) and FBs are not an exception. Furthermore, effective communication does not only contribute to improved productivity, but also leads to greater satisfaction, motivation and increased morale of IC in FB, and thereby their level of commitment to the organization. For FBs, the use of formal communication channels is less frequent than NFBs (Pittino and Visitin, 2013).

After reviewing the literature, we presented the following research questions. How does the IC behave in the FB? Which are the main strategies and actions undertaken by the FB with respect to its ICs? And finally, do different perceptions regarding distinct agents implied in the study and management of the FB exist?

## Methodology

### Design

The research was carried out through semi-structured, in-depth interviews, as these are highly effective for understanding what people think or believe about certain aspects (Krueger and Casey, 2014). In this way, the objective of identifying the main characteristics and behavior of IC in FBs, and the strategies and actions that successful FBs undertake to get greater satisfaction was achieved. Afterward, information was analyzed through two procedures, depending on the data obtained. One was focused on the quantitative analysis through the frequency analysis and contingency tables, and the other was qualitative analysis of the interview information (Braun and Clarke, 2006). One of the recommendations of the literature when this methodology is used is to select candidates who are truly qualified in the area of study, and to recruit them until a point of saturation is reached at which no new information appears (Krueger and Casey, 2014). The use of this methodology could also be justified because it is a little known subject as established Morse and Richards (2002). Otherwise, Yin (1994) recommends the use of this methodology to analyze a situation that has been studied infrequently and that is unique, and we can learn something new and important. By that, he establishes a protocol to justify the validity and reliability of the qualitative analysis that we have followed in this research.

### Participants

Purposive sampling was undertaken for the selection of participants. The most important factor was selecting specific participants who were the most prepared to provide the researcher with the best understanding of the issue being analyzed and to respond to the object of this research in the best way possible. Ethical approval was not required for this study in accordance with the national and institutional guidelines. Firstly, experts were selected from outside of FB such as professors, researchers and consultants of this type of business, all belonging to the FB department of the Scientific Association of Economics and Business Management (ACEDE)^1^. Secondly, managers and employees who were or were not family members, belonging to different FBs were sought. The recruitment was stopped when a point of saturation was reached in the data collected. Ultimately, the sample consisted of 31 participants, of whom 14 were professors, researchers or consultants of FBs, 6 were family member managers, and 11 non-family member employees of FBs. The sample thereby contained interviewees who were well-qualified, although from different backgrounds and with different experiences, so that the sample was sufficiently diverse. Of the 31 participants, 17 were men and 14 were women, with ages spread in the following way: 9.7% under 30; 29% between 31 and 40; 45.2% between 41 and 50; 12.9% between 51 and 60%; and 3.2% over 60 years old.

Different works which use a similar methodology use similar or even smaller samples (Galea et al., 2014; Lyddy and Good, 2016; Mehra et al., 2016).

#### Data Collection

The data was collected between November 2016 and January 2017, and all participants were informed of the confidentiality of information and the purpose of the work, all-agreeing to participate in it. When possible, interviews were conducted in person, but due to logistical issues, some were conducted by telephone. The 31 participants were guided with semi-structured interviews, with a list of topics that served as a guide, following the instructions set out in the literature (CBO, 2004). The list of topics includes general aspects aimed toward the IC, influential psychological factors, actions undertaken in recruitment and selection of employees, training and development, evaluation and remuneration systems, and participation and communication of the IC. The guide of the interview includes both open and closed questions and a panel of experts with different experiences reviewed it before being carried out. One of them was a researcher and another was a senior manager of a FB. In this way, we contrasted the validity of the interviews (Yin, 1994).

The interviews were carried out in Spanish, as all were intended to be carried out in a common language. Furthermore, both the interviewer and the interviewee are highly qualified and this facilitated understanding of thoughts and ideas. A special emphasis was placed on there being no right or wrong answers and that the interviewer equally valued all opinions. 73% of the interviews lasted between 20 and 30 min, and the remaining 27% lasted longer than half an hour.

Once the interview was completed, the researchers analyzed whether new information had been collected or whether the point of saturation had been reached, thereby determining when to proceed to analysis of the data. Once the interviews were transcribed, the information from the closed questions was statistically analyzed, while the information from the open questions was encoded and the details of each category found was analyzed. Two researchers undertook this encoding separately, and the different classifications were analyzed until a consensus was reached. Only in the case of not reaching consensus did a third party intervene to decide the most appropriate category in which to include it. In this phase, special emphasis was placed on the ability to analyze the questions and the specific meaning of the words and phrases that seemed most significant (Corbin and Strauss, 2008). Although some experts (Morse and Richards, 2002) argue that a single researcher conducting all the coding is both sufficient and preferred, this could be particularly true in studies where being embedded in ongoing relationships with research participants but not in this case.

## Results

### Quantitative Analysis

To analyze the information collected from the interview through the closed questions, a frequency analysis was carried out, which was very useful for describing the opinions with regard to the issues raised.

For the case of characteristics that shown by ICs of FBs, based on the opinion of the participants in in-depth interviews, the following data was found:

– 38.7% of participants establish that the main motivation of the IC is transcendent (altruistic vision), 35.5% intrinsic, and just 25.5% determine it to be extrinsic motivation.
– With regard to the role of women in FBs, 58.1% of participants determine that there is no difference with regard to FBs, if we compared with 35.5% who establish that the role is more important in FBs, and just 6.5% who establish the role to be less important in FBs.
– 45.2% consider ICs to have greater social intelligence, compared with 41.9% who consider it the same level as in NFBs, and 12.9% who consider it lower.
– The use of work teams is considered by 51.6% as the same in NFBs, although 22.6% consider its use greater in FBs, and the remaining 25.8% consider it less.
– Nepotism is seen by 61.3% as a risk to the FB, although 35.5% do not consider it negative or positive.
– The diversity of teams is considered greater in FBs in 48.4% of cases, compared with 51.6% who consider diversity greater in NFBs.
– Leaders selected for their qualities are considered more prepared than leaders who inherit their job in 80.6% of cases.
– Finally, relationships with greater problems may arise in FBs if they are conflicts between siblings (61%), between cousins (41.9%), between parents and children (25.8%) and finally between married couples (21%).

When we analyze the strategies and actions undertaken by FBs to increase the satisfaction of their ICs, we find the following results:

– Recruitment and selection processes seek alignment between the employee and the job in 64.5% of cases, compared with 35.5% who consider the FB to seek alignment between the employee and the values of the organization.
– The selection criteria used are based on measurable standards in 74.2% of cases, compared with 25.8% who perceive blood relationships as the main selection criteria in FBs.
– 51.6% establish that the FB is more oriented toward job training, while 45.2% consider it oriented more toward the development of long-term professional careers.
– With regard to the remuneration system, 29.0% consider remuneration in FBs to be above market levels, compared with 22.6% who consider it below market levels. 45.2% establish that the remuneration system is essentially non-monetary, compared with 12.9% who consider it solely and exclusively based on monetary aspects. 51.6% consider the salary used by FBs to be fixed, compared with 22.6% who consider variable salaries to be used more.
– 51.6% establish that the evaluation system the FBs use mainly measures qualitative type aspects and is not formalized, compared with 48.4% who believe the opposite.
– The participation and communication mechanisms of FBs are considered formal in 54.8% of cases, compared with 45.2% in which they are not.
– Finally, 87.1% establish the issue of making the FB professional a priority for obtaining greater satisfaction among their ICs, compared with 12.9% who do not consider it a priority.

As we can observe from the information obtained from the interviews, there is not a consensus on the main characteristics of the IC or on what are the best mechanisms for generating greater satisfaction among them. For this reason, we have proceeded to analyze this information through contingency tables in order to determine whether there is a relationship between these opinions and the characteristics of the participants. Specifically, we have analyzed the contingency tables of each one of the questions analyzed, and the type, gender and age of the participant. This type of statistical analysis does not provide us with either the magnitude or direction of the association between the variables, therefore in the case of finding association between them, we have proceeded to analyze the contingency coefficient to discover these dimensions.

With regard to the age and gender of the participants, we must state that we found no relationship between their responses, but the same does not occur when we analyze the relationship with the participant type variable. In this case, the information obtained after contingency analysis is that there is a statistically significant association between the type of participant variable and the majority of variables studied as IC characteristics, as we can observe in **Table 1**.

**Table 1:** Association between variables of the study with regard to the characteristics of the IC.

Variable	Statistical association with regard to the type of participant^∗^	Chi squared (degrees of freedom)	Relationship between variables. Contingency coefficient
Types of IC motivation	57.1% EE and 50% FM – intrinsic motivation 72.7% NFE – transcendent Motivation	χ^2^(4) = 11.846 *p* = 0.019	High and directly proportional C = 0.526

Importance of women in the FB	57.1% EE – Equally important 50% FM and 90.9% NFE – Less than in non-FBs	χ^2^(4) = 9.024 *p* = 0.061	Moderate and directly proportional C = 0.475

Social intelligence	64.3% EE – Lower in the FB 50% FM – Equal 72.7% NFE – Greater in the FB	χ^2^(4) = 14.275 *p* = 0.006	Moderate and directly proportional C = 0.562

Nepotism	78.6% EE and 83.3% FM – Perceived as a risk 72.7% NFE – Not perceived as a risk	χ^2^(4) = 11.121 *p* = 0.025	Moderate and directly proportional C = 0.514

*^∗^EE, external experts (professors, researchers, and consultants); NFE, non-family employees; FM, family member managers*.

As shown in **Table 1**, there is not a consensus on the characteristics of the IC in the FB. Depending on the group asked, we find different profiles.

EE group (78.6%) see nepotism as a great risk to the FB, and although they consider the predominant motivation among ICs to be intrinsic motivation (57.1%), they understand that they are less skilled in terms of social intelligence (64.3%) and that the roles that women play is of equal importance as in non-FBs (57.1%).

FM group evaluates nepotism similarly as a risk factor (83.3%) and intrinsic motivation as being predominant (57.1%); however, they do not find a difference in the roles of women in FB (50%) or in the degree of social intelligence of IC (50%).

With regard to NFE group, we can observe different perceptions. This group considers the main motivation to be transcendent (72.7%), the roles of women to be lesser than in non-FBs (90%), the IC having greater social intelligence (72.7%) and not perceiving nepotism as a risk (72.7%).

In the same way, we find an association between the participant type variable and some of the variables analyzed on strategies and actions carried out by the FB to improve IC satisfaction, as we can observe in **Table 2**.

**Table 2:** Association between variables of the study with regard to strategies and actions the improve IC satisfaction.

Variable	Statistical association with regard to the type of participant^∗^	Chi squared (degrees of freedom)	Relationship between variables. Contingency coefficient
Recruitment and selection	61.5% EE – FBs seek alignment between the individual and the organization 100% FM and 81.8% NFE – FBs seek alignment between the individual and the job	χ^2^(4) = 9.944 *p* = 0.041	Moderate and directly proportional *C* = 0.494

Selection process	50% EE – Blood relations predominant in selection 100% FM and 90.9% NFE – Standardized and measurable criteria used	χ^2^(2) = 7.972 *p* = 0.019	Moderate and directly proportional *C* = 0.452

Remuneration policy (I)	77.8% EE – Salaries lower than market 100% FM and 100% NFE – Salaries higher than market	χ^2^(2) = 9.679 *p* = 0.008	High and directly proportional *C* = 0.614

Remuneration policy (II)	100% EE and 66.7% FM – Remuneration mainly non-monetary 50% NFE – Remuneration mainly no monetary	χ^2^(2) = 5.464 *p* = 0.065	Moderate and directly proportional *C* = 0.483

Evaluation system	85.7% EE – Mainly qualitative, non-formalized criteria 66.7% FM and 50% NFE – Mainly quantitative and formalized criteria	χ^2^(2) = 12.245 *p* = 0.002	High and directly proportional *C* = 0.532

Participation and communication	64.2% EE – Use of informal channels 50% FM – Use of formal channels 81.8% NFE – Use of formal channels	χ^2^(2) = 5.357 *p* = 0.069	Low and directly proportional *C* = 0.384

*^∗^EE, external experts (professors, researchers, and consultants); NFE, non-family employees; FM, family member managers*.

As seen in **Table 2**, the strategies and actions that participants have established as those, which improve the satisfaction of ICs in FBs also differ between the groups from which we have obtained the information.

EE group believes that the selection of staff must seek alignment between the individual and the organization (61.5%), salaries must be below those of the market (77.8%), remuneration should incorporate non-monetary aspects (100%), evaluation systems should be focused more on qualitative and non-formalized criteria (85.7%) and more informal communication channels should be used (64.2%). Meanwhile, the FM and NFE groups have a different perception, more oriented toward the professionalization of the FB, seeking greater alignment between the individual and the job (100% FM and 81.8% NFE), standardizing and measuring selection criteria (100% FM and 90.9% NFE), salaries above those of the market (100% FM and 100% NFE), with remuneration that incorporates non-monetary aspects (66.7% FM and 50% NFE), the use of quantitative and formalized evaluation criteria (66.7% FM and 66.7% NFE) and improvement of formal communication channels (50% FM and 81.8% NFE).

### Qualitative Analysis

As previously explained, the interviews were transcribed for subsequent study. From the encoding of the interviews carried out, we obtained a first result in which we checked that the information obtained based on the participants differing from each other, as occurred in the quantitative analysis. Below (see **Table 3**), we show the encoded results and select narrative bullet points, derived from the constant comparison between the texts, as suggested by Suddaby (2006). All qualitative information collected was analyzed following the theory approach (Corbin and Strauss, 1990) in which the main aspects are classified (Riessman, 1993).

**Table 3:** Information from the qualitative analysis.

Topic	Encoding^∗^	Narrative bullet point
How the FB generates satisfaction and cooperative behavior between its ICs	EE-generating trust, alignment of objectives, commitment of employees, participation in decision making	“When everyone understands responsibility, effort and the benefit of working together as a family, always taking into account the common good and minimizing personal gain”
	
	FM-professionalization, separation of family and business	“Properly outlining the roles of each member of the business, separating family relationships from working relationships as far as possible”
	
	NFE-encouraging communication and personal relationships	“With friendly, proper relationships with employees, allowing all members involved in all decisions”

Mechanisms of the FB for attracting, retaining, motivating and developing their ICs	EE-use of informal mechanisms, emotional management, avoiding nepotism	“FBs use more informal mechanisms, which are equally effective as NFBs. They use emotional aspects and offer a more stable bond in the long term”
	
	FM-professionalization of FB	“FBs tend to have a greater professionalization of talent retention and development processes. However, there is still a significant gap between FBs and NFBs in this regard”
	
	NFE-use of the same mechanisms as non-FBs but more focused on IC	“They use the same mechanisms as non-FBs, but more focused on the employee as your job in a FB becomes like a second family in some way, so when you have worked for the business for some time you are more involved than in a NFB”

Most influential characteristic in FBs	EE-participation in decision making and continuity	“Continuity is a relevant aspect in FBs, which results in the preservation of ownership through the participation of the best professionals of the family”
	
	FM-continuity	“Continuity is a relevant factor as the founders place more emphasis on this when they foresee continuity. In simple terms, parents strive more for the business if their children will continue in it.”
	
	NFE-ownership and continuity	“They care more about the progress of the company, as they own it.” “…they place a greater emphasis on obtaining new customers and care more about ICs than a non-FB because they want to transfer it to their children”

Competitiveness of the FB	EE-less competitive than non-FBs	“Less competitive in terms of economic gain, they are also less productive; nevertheless, they pursue and obtain non-economic advantages that complement their level of satisfaction”
	
	FM-more competitive if they professionalize	“They are more competitive if they professionalize. When in the added value of the commitment of individuals and the strength of working on the same project together is added to professionalism, it makes the FB more competitive”
	
	NFE-equal or less than non-FBs. Organizational commitment	“I believe that in general they are equally or less competitive because they do not choose the best individuals for each job, although they achieve good results due to having personnel who are more committed to the business”

*^∗^EE, external experts (professors, researchers, and consultants); NFE, non-family employees; FM, family member managers*.

**Table 3** shows some similar issues to those obtained in the quantitative analysis as EE group establishes trends related with the alignment of objectives, the use of more informal mechanisms and the achievement of gains which are not strictly economic as main differentiating factors of FBs which must be incentivised to achieve greater IC satisfaction. However, it is clear that the main aspect that FM group highlight is the orientation toward professionalism as a way to improve the cooperative behavior, motivation and satisfaction of the IC. Finally, NFE group shows a clear orientation toward the IC, focused on personalized relationships, communication, participation in decision making and commitment.

#### Research Findings

A number of conclusions have been reached. First, we find a greater motivational quality among ICs of FBs. Although literature establishes intrinsic motivation as predominant in FBs (Tyler and De Cremer, 2006), according to non-family members employees, FBs show more importance on the transcendental motivation based on altruistic behavior. This motivational quality is shown in more personal and integrated relationship among members of FB, relatives or not. However, we were not able to reach consensus on qualities such as greater social intelligence, diversity of work teams, or the role of woman in these companies, which evolutionary theory assume (Nicholson, 2008).

According to nepotism, that literature has always seen it as a great risk to FB (Dyer, 2006), the FM group also considered it as a risk. In contrast, the NFE group do not perceive it as such. A possible explanation is that although they are aware that it may exist, they do not perceive it as an element that may have negative consequences either for themselves or for the survival of the business. Conversely, the fact that business remains within the family is a guarantee of continuity, due to the responsibility and effort that the managers show with the main economic support of the family, both in the present and the future.

Some similar occurs about internal conflicts among family members. Conflicts among siblings have been as the greatest risk to FB and it decreases when conflict is among cousins or among parents and children.

Another great aspect to consider is the disparity found both in quantitative and qualitative analysis regarding strategies and actions to be undertaken by the FB to allow the satisfaction of the IC, and thereby its success. First, the EE group focuses more on the development of more informal aspects related with the management of emotions and trying to extend qualitative and personal type aspects. Some studies establish that emotions can limit the firm in its ability to adapt the certain business demand (Vandekerkhof et al., 2015). By that, we put emphasis on develop emotions theory, if we want preserve of socio-emotional wealth and help FB to keep family control without it do not affect negatively the decision-making process.

Second, the FM group focuses on professionalizing the business and try to continue the same development that non-FBs. Sánchez-Marín et al. (2017) established that more formalized human resource practices, increase the financial performance of the company. Despite the fact that FB put more emphasis on non-formalized human resource practices adapted to non-economic goals related to the welfare of the family and employees.

And finally, the NFE group is the only one, which establishes that orientation toward the IC must be the essential pillar for achieving these objectives. This reinforced the idea that the impact of some practices on IC satisfaction create comparative advantage (Ullah and Yasmin, 2013). This support the basic premise that if employees in FB receive greater support from FB, they are like to reciprocate with greater affective commitment and greater efforts to contribute to achieving organizational performance (Huang, 2013).

From an integrated vision, we can establish the essential criteria that the FB must maintain a balance between these three dimensions. Professionalizing the business is essential, but FB must do so by integrating qualitative and emotional aspects into its management, with special orientation toward to IC. In this way, creating more personable organizations, which do not lose their orientation toward the market, but which are able to make decisions in which certain short-term economic objectives are renounced in order to strengthen the future competitive advantage of the business.

## Discussion, Academic Implications, Limitations, And Future Research Lines

Although we found much literature in the field of FB, few studies empirically analyze its issues and focus on the IC. The initial research was based on psychological theories, which have been applied to FBs in recent years (Nicholson, 2008; Pieper, 2010). It contributes to focus on the research of FB from others points of view. Even then, psychology has a lot to offer to better understanding FBs and their particular behavior (Pieper, 2010).

Scholar have not paid enough attention on IC, despite the fact that individual human capital in FB is often inferior to that non-FB (Dawson, 2012) and FBs have difficulties managing their human resource, especially when it concerns a family members (King et al., 2001).

This paper is a response to the need for further research on this line to increase the knowledge about IC behavior, highlighting the strategies and actions that FBs implement to improve their satisfaction.

To that end, we build on a combination of evolutionary psychology (Björnberg and Nicholson, 2007) and SEW perspective (Berrone et al., 2012) to explain why FBs need use different practices to increase IC satisfaction. And we collected data from 31 participants with different backgrounds and experiences (experts, family business managers, and non-family members employees) to try to understand some important gaps and contradictions that there are still in literature on FB (Sánchez-Marín et al., 2017).

This paper has also academic implications for IM literature contributing new evidence to the importance academic debate about the effectivity of some practices in FB in terms of IC satisfaction. Furthermore, this study offers several contributions from the viewpoint of business practices. First, the paper emphasize that IC orientation gets a balance between economic goals and non-economic goals like continuity and preservation of family wealth (Sánchez-Marín et al., 2017). Considering that the main goal of the society is to create competitive and sustainable companies, FB should be seen like a more flexible structure, with a whole vision of the needs of company’s members and thus, develop a motivational quality about their members. It makes we think to regards as more human organizations, whenever FBs do not fall into several traps like an excessive nepotism, the lack or insufficient professionalization in the decision-making process or the loss of employees orientation as members of the company and family at the same time.

Non-family businesses can learn about FB to develop knowledge about emotions management, because it could help to develop a better workplace and a friendly climate and it would have an impact on the productivity and innovation of the company (Bammens et al., 2015).

Finally, this study is not without limitations, which may provide fruitful lines for future research. It would be desirable to include more participants in the research, using explicit measures for each dimension such as professionalization, motivational quality and IC orientation. It would provide more information about different types of FBs and the interrelation among variables and its effect on firm performance and continuity of FB.

In short, this study finds that an integrated vision and a balance among different objectives are needed in FB. According to experts, family managers and non-family employees, a balance among management emotions, professionalization and IC orientation are needed to reach the continuity of FB.

## Author Contributions

SG-B and PJ-E played an important role in the design of the study. PJ-E and MZ-B took charge of carrying out the in-depth interviews and the processing of data. SG-B and MZ-B analyzed and interpreted the data. All authors reviewed the article and gave the manuscript their final approval.

## Conflict of Interest Statement

The authors declare that the research was conducted in the absence of any commercial or financial relationships that could be construed as a potential conflict of interest. The reviewer MR and the handling Editor declared their shared affiliation, and the handling Editor states that the process nevertheless met the standards of a fair and objective review.

## Footnotes

1 ACEDE is an association of university professors interested in improving scientific research on the main issues arising from business administration and disseminating this knowledge between both students and managers. It was created in Spain in 1990. More information can be found on its website, at http://www.acede.org/.
